# Isolated localization of Rosai Dorfman disease as renal mass: a case report and review of literature

**DOI:** 10.11604/pamj.2016.24.64.6291

**Published:** 2016-05-13

**Authors:** Aziz El Majdoub, Aziza El Houari, Laila Chbani, Hinde El Fatemi, Abdelhak Khallouk, Moulay Hassan Farih

**Affiliations:** 1 Department of Urology, Hassan II Hospital University Center, Fez, Morocco; 2 Laboratory of Pathological Anatomy and Cytology, Hassan II Hospital University Center, Fez, Morocco

**Keywords:** Rosai dorfman disease, renal mass, extranodal, emperipolesis

## Abstract

We report a rare case of an elderly woman presented with right renal mass with invasion of renal vein and several small lymphadenopathy in the hilar area. The diagnosis of kidney cancer is suspected and the patient underwent open radical nephrectomy, surrenalectomy and lymphadenectomy dissection. The pathologic examinations find a rosai dorfman disease. This unusual benign entity is uncommon in the kidney, but in medical imaging, it may simulate an infiltrative renal neoplasm, especially a lymphoma or leukemia or even renal cell carcinoma. A comprehensive literature review was undertaken to summarize the clinical and pathologic features of this disorder.

## Introduction

Rosai-Dorfman disease (RDD) or sinus histiocytosis is a rare benign disease of unknown etiology. Classically, it is characterized by lymphadenopathy mainly in the cervical area associated with fever, leukocytosis, polyclonal gammopathy, and an elevated erythrocyte sedimentation rate. Histopathologically an inflammatory proliferation of S 100 protein-positive histiocytes characterized by lymphophagocytosis is seen. Most cases involve patients in their first and second decades, but all age groups can be affected [1, 2]. Extranodal sites are involved in 43% of cases, and lymphadenopathy may be absent in such patients [3]. We report a case of a woman with a Rosai Dorfman disease presenting as extranodal renal mass. We review the clinical presentation and essential histopathologic features necessary for the accurate diagnosis of this disease.

## Patient and observation

**Figure 1:**
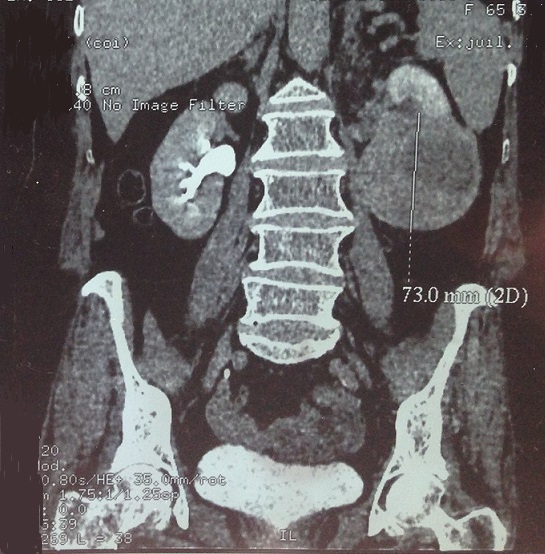
Abdominal and pelvic computed tomography scan showed the presence of a large tissue process at the upper pole of the right kidney measuring 8 cm of diameter, demonstrating heterogeneous contrast enhancement

**Figure 2:**
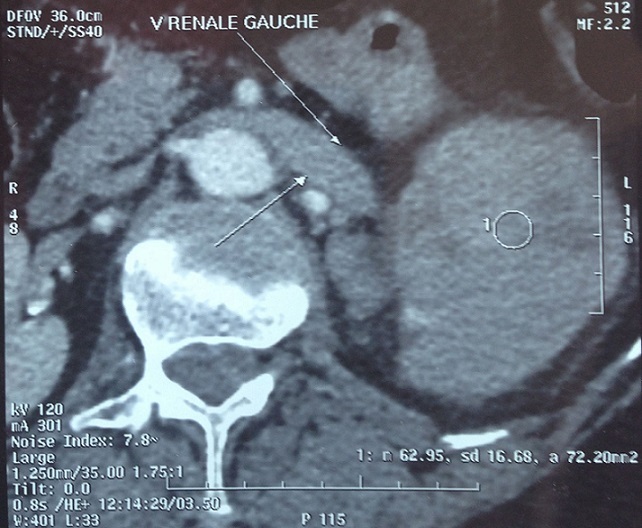
Abdominal and pelvic computed tomography scan showed that the process extends over the hilar area with invasion of the right renal vein, while the lower vena cava is permeable

**Figure 3:**
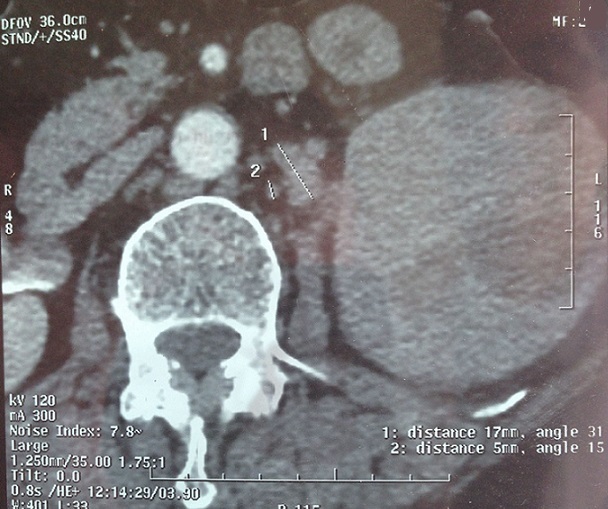
Abdominal Computed tomography scan showed Several small lymphadenopathy in the right hilar area

**Figure 4:**
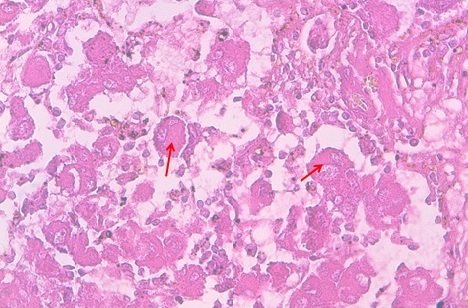
High-power magnification demonstrating histiocytic proliferation with intracytoplasmic lymphocytes (emperipolesis, arrows), Hematoxylin-eosin stain, original magnification x40

**Figure 5:**
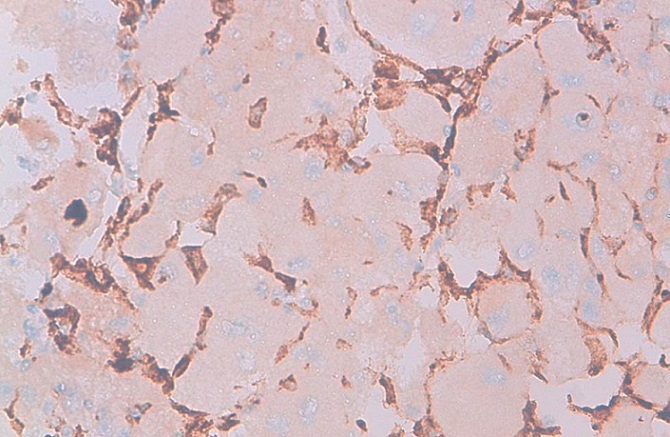
High-power magnification with cytoplasmic immunostaining of histiocytic marker (CD68) highlighting proliferating cells (CD68 stain, original magnification x40)

68-year-old woman presented with hematuria and right flank pain since 3 years. The patient had no history of smoking and no other medical problems. The physical examination revealed a sensitivity of a lumbar fossa and had not revealed any adenopathy, fever or weight loss. Laboratory examinations were normal. The abdominal and pelvic Computed tomography scan showed the presence of a large tissue process at the upper pole of the right kidney measuring 8 cm of diameter, demonstrating heterogeneous contrast enhancement.this process extends over the hilar area with invasion of the right renal vein, while the lower vena cava is permeable. Several small lymphadenopathy in the right hilar area (Figure 1, Figure 2, Figure 3). There is no other abdominal pelvic lymphadenopathy. Diagnosis of renal cell carcinoma of the right kidney with hilar nodal metastases is suspected. She underwent open right radical nephrectomy with surrenalectomy and regional lymph node dissection. The operation was performed without complications. The final histological examination of the renal mass and regional lymph node showed “emperipolesis”, which is characterized by intact inflammatory cells in the cytoplasm of large histiocytes (Figure 4). Histiocytes were positive for CD-68 and negative for CD10, anti CKE1/AE3, antiCK8/18, anti CK19, antidesmine, antiHcaldesmone, CD56, anti synaptophysine, antivimentine and antiMelanA (Figure 5). These findings are typical characteristics of Rosai–Dorfman disease and eliminate others diagnosis.

## Discussion

Rosai-Dorfman disease or sinus histiocytosis with massive lymphadenopathy was first described in 1969 by Rosai and Dorfman. It is classified as a non-neoplastic histiocytic disorder and is classically characterized by massive lymphadenopathy secondary to infiltration and dilation of the lymph node sinuses by large histiocytes, mainly in the cervical area.

Generally manifests in children or young adults with massive cervical lymphadenopathy, fever, leukocytosis, an increased erythrocyte sedimentation rate and hypergammaglobulinemia. Other lymphatic groups such as mediastinal, axillary and inguinal lymph nodes can also be affected [2]. The etiology is unknown, although an infectious or chronic inflammatory cause has been implicated. It is also known that clinically significant immunologically mediated disease can occur in association with sinus histiocytosis with massive lymphadenopathy and may adversely affect the prognosis. Extranodal disease has been reported in 43% [3]. Kidney involvement is very uncommon, and therefore sinus histiocytosis is not frequently considered in the differential diagnosis of an infiltrative renal mass.

To our knowledge there only four cases of RDD in kidney cases have been reported with two of them diagnosed with adenocarcinoma of the prostate. In a rare case reported by Buchino et al, the kidney was only focally involved with a single small mass in the lower pole that contained an admixture of histiocytes, lymphocyte and plasma cells [4]. In another case reported by Bechtold et al, a lobular irregularly enlarged kidney with distorted calyces associated with large matted para-aortic lymph nodes was described and the diagnosis was RDD [5]. Associated symptoms and signs may be caused by specific organ involvement or may be constitutional, such as fever and weight loss. Laboratory findings include anemia, leukocytosis and serum polyclonal hypergammaglobulinemia. The histological findings in extra-nodal RDD are characterized by dense infiltrate of histiocytes with scattered lymphocytes, plasma cells, and neutrophils. The histiocytes are larger in size with large vesicular nuclei, small nucleoli, and abundant pale pink cytoplasm. Emperipolesis (the presence of intact lymphocytes, plasma cells, neutrophils, and red blood cells) within histiocytes is the pathognomic feature [6]. Immunohistochemistry shows the positivity for S-100 and CD68 and the negativity for CD1a [7].

The differential diagnosis of RDD in kidney includes malignant fibrous histiocytomas and histiocytic proliferations of infectious etiology (the presence of S100 is useful in discriminating these lesions), leukemia or lymphoma, especially when accompanied by lymphadenopathy (absent of emperipolesis and Immunohistochemistry profiles help to correct diagnosis). Other possible differential diagnoses include storage disease, tuberculosis or even renal cell carcinoma, a metastatic tumor such as malignant melanoma. On the basis of the sonographic and CT appearances, we could not differentiate this entity and biopsy was recommended to exclude those diagnosis. The clinical course varies widely from spontaneous remission to death from vital organ infiltration. Patients with renal involvement have been associated with poorer outcomes, with 40% of patients dying of the disease and the remainder having persistent involvement [2, 8]. A definitive treatment regimen has not been established, but a combination of chemotherapeutic agents and corticosteroids appears to be the most effective [9]. Surgery is reserved only for those with compromise of adjacent vital organs by the disease. To our knowledge, this represents the second reported case of extranodal variant Rosai-Dorfman disease presenting as a renal mass in an elderly patient without a prior history of the nodal variant disease.

## Conclusion

Rosai-Dorfman disease of the kidney, although rare, should be considered in the differential diagnosis of renal mass especially when it is bilateral.in computed tomography scan The appearance can resemble renal cell carcinoma and lead to radical nephrectomy.
